# Nutrition Practices among Adult Cancer Survivors Living on the Island of Ireland: A Cross-Sectional Study

**DOI:** 10.3390/nu14040767

**Published:** 2022-02-11

**Authors:** Niamh O’Callaghan, Pauline Douglas, Laura Keaver

**Affiliations:** 1 Department of Health and Nutritional Science, Institute of Technology Sligo, Ash Lane, F91 YW50 Sligo, Ireland; niamh.ocallaghan@mail.itsligo.ie; 2 Nutrition Innovation Centre for Food and Health (NICHE), School of Biomedical Sciences, Ulster University, Coleraine BT52 1SA, UK; pl.douglas@ulster.ac.uk

**Keywords:** cancer survivor, nutrition, diet quality, food choice, supplement

## Abstract

The purpose of this research was to explore the nutrition practices among post-treatment cancer survivors across Ireland. Cancer survivors aged 18+ years living across Ireland, who were not palliative and had completed active cancer treatment at least six months previous, were recruited to complete an online survey assessing dietary quality, food choice and satisfaction with food-related life as well as clinical and nutrition status. It was circulated by cancer support networks and on social media. Descriptive statistics are presented. The cohort (n = 170) was predominantly female (85.9%) and had breast cancer (64.7%). Mean age was 51.5 ± 10.9 years and 42.7% of the cohort were >five years post-treatment. Only 20% and 12% of the cohort had been assessed by a dietitian during and post-treatment, respectively. The mean dietary quality score was 10.3 ± 1.7, which was measured by the Leeds short-form food frequency questionnaire (SFFFQ). Using a 5-point Likert scale, the median satisfaction with food-related life score was 19 (3.3), which evaluates cognitive judgements on the person’s food-related life. The food choice questionnaire (FCQ) assesses the relative importance of a range of factors related to dietary choice to individuals. The primary determinant of food choice in this cohort was the natural content (31.7%) followed by health (24.7%). Vitamin and mineral supplement use was reported by 69.8% of the cohort; the most consumed was Vitamin D. Four themes emerged from an optional open-ended question: awareness of nutritional importance; desire for specific nutritional advice and dietetic referral; cancer and treatment nutrition impacts were highlighted; as well as struggles with weight gain. This research provides useful insight into the nutrition practices of Irish cancer survivors. A desire and need for individualised and specific advice are evident.

## 1. Introduction

In Ireland, there are more than 200,000 individuals living with or beyond cancer, equating to almost 4% of the total population [1]. In the next 25 years, this figure is expected to double due to demographics, earlier detection, and improved treatment outcomes [1]. The need to recognise the individual and distinct needs of cancer survivors throughout Ireland has been highlighted by the government through the development of the National Cancer Strategy 2017–2026 [2]. For this study, the definition from this strategy will be utilized, where “a cancer survivor is a person with any type of cancer who has undergone treatment, completed the intervention and is living” [2]. The strategy emphasizes how cancer survivorship is a distinct phase of the cancer journey and how the needs of cancer survivors have not been prioritised to date. The supporting documents of this strategy include a report titled the *Acute Sector Cancer Survivorship Services in the Irish Context* from the National Cancer Control Programme [3] and a scoping review by both the National Registry and the Irish Cancer Society [4]. Evidently, survivorship care is increasingly becoming a national priority; these publications highlight several unmet needs of cancer survivors and confirm the lack of nutritional data in this population.

Nutrition is an important aspect of care in the management of cancer at all stages, from diagnosis to survivorship [5,6]. An individual’s transition from a cancer patient to a cancer survivor is challenging and often associated with lifestyle and behavioral changes, including those of a nutritional nature. Often side effects of treatment can persist, such as fatigue, taste, and smell changes. This can negatively impact the ability or desire to consume food and partake in physical activity [7]. Literature shows adherence to a higher intake of vegetables and fish as well as a prudent dietary pattern is inversely associated with overall mortality among cancer survivors, whereas a Western dietary pattern is positively associated with overall mortality in this population [8]. In comparison to the general population, studies show consumption of a diet of poor quality, with low fruit and vegetable intake among cancer survivors [9], and low participation in physical activity [10,11]. Cancer survivors can experience unwanted weight gain, especially breast cancer survivors [12]. Furthermore, survivors are also at increased risk of chronic conditions such as diabetes, osteoporosis, and cardiovascular disease [13]. Diet is an important modifiable factor that could reduce these risks, thereby promoting their long-term health [14]. 

Irish literature in this area is limited, with few studies focusing on post-treatment nutrition status. In a study carried out by Timon and Doyle on the nutrition support needs of Irish cancer survivors (total cohort n = 76), 65% of the cohort who were one-year post-treatment reported still facing dietary issues because of their cancer and treatment [15]. Of the respondents, 23% (n = 17) felt their current diet was worse now than prior to their cancer diagnosis and had a desire for dietary support relating to practical information on how to achieve the recommended nutrition guidelines. In a recent national survey of cancer survivors carried out by Sullivan et al. (2021), nutrition was rated very important by 89% of the total cohort with only 39% of the cohort getting referred to a registered dietitian. Additionally, over half (57%) of the respondents felt confused by the often-conflicting nutrition information available in the media and offered by people around them. In this study, most respondents were still receiving treatment [16]. 

Cancer survivors cite nutrition as being important [16,17,18,19] and feel it should form a fundamental part of the cancer care continuum; that said, this is not always the case. In Ireland, oncology dietetic services in both the public and private healthcare systems are severely under-resourced [20]. In addition, despite the availability of international guidelines for nutritional support in an oncology setting, the approach toward this issue varies considerably among medical oncologists [21]. In consequence, insufficient professional advice coupled with a desire for information may lead some cancer survivors to seek out information about diet themselves. When searching in popular media or online, cancer survivors encounter a wealth of information, not all of which is reliable or accurate [22]. There is an abundance of media misreporting of the dietary factors that are linked to cancer risk that could be misleading, particularly if they believe the sources to be trustworthy [23]. 

Survivorship care providers and policy makers need a better understanding of the nutrition practices among cancer survivors to address relevant issues and to improve and target care. This study, therefore, aims to describe the nutrition practices among survivors who are post active treatment in Ireland; in particular, looking at dietary quality, food choice and satisfaction with food-related life. A further aim is to determine vitamin and mineral supplement use, post-treatment food changes and sources of dietary advice among cancer survivors.

## 2. Materials and Methods

### 2.1. Study Design 

This was a descriptive, cross-sectional study, and we collected data in the form of an online questionnaire exploring the nutrition practices among cancer survivors living across Ireland. 

### 2.2. Inclusion Criteria 

Inclusion criteria for recruitment included cancer survivors living on the Island of Ireland, aged 18+ years, who were not palliative and had completed active cancer treatment at least six months ago. 

### 2.3. Recruitment 

Recruitment took place from the start of October 2020 to mid-December 2020. Cancer survivors were recruited via emails circulated by cancer support networks across Ireland and online through social media platforms (Twitter/Facebook). 

### 2.4. Ethical Approval 

Ethical approval for this study was provided by the Research Ethics Committee in the Institute of Technology Sligo, reference number: 2020023. 

### 2.5. Questionnaire 

The questionnaire was housed in Microsoft Forms; it had five sections and the average time to complete the form was 18 min. The form was piloted with 6 academics. Overall, they felt the questionnaire was clear and easy to understand and complete. The full version of this questionnaire is provided in the Supplementary Materials File S1. 

#### 2.5.1. Demographic, Clinical and Nutrition Characteristics 

Participants were asked about their age, gender, highest level of educational attainment, employment status and current living arrangement. Questions on cancer diagnosis included year of diagnosis, previous treatments received and how long ago they had completed treatment. Participants were also asked to report their weight (kg) and height (m^2^) and if they had experienced any fluctuations in weight in the last six months. Nutrition questions focused on supplement use, post-treatment food changes, access to a registered dietitian and source of dietary advice. 

#### 2.5.2. Food Frequency Questionnaire 

The Leeds short-form food frequency questionnaire (SFFFQ) was utilized to measure dietary quality and estimate the dietary quality score (DQS) [24]. This questionnaire includes 25 food items and focuses on fruit, vegetables, fibre-rich foods, high-fat and high-sugar foods, meat, meat products and fish. It evaluates dietary quality over the past month. The tool was validated in 2016 on a UK population [24] and is accompanied by an Excel spreadsheet that calculates the DQS, which was used for this study. These calculations are based on an estimate of what the frequency options would equate to in portions per day, multiplied by an average portion size for each food item giving an estimate of what, on average, each frequency option equates to in grams per day for each SFFFQ item. The minimum DQS is 5 and the maximum, indicating optimum dietary intake for these foods, is 15.

#### 2.5.3. Food Choice Questionnaire 

The food choice questionnaire (FCQ) developed by Steptoe, Pollard and Wardle in 1995 was designed as an instrument to assess the relative importance of a range of factors related to dietary choice to individuals [25]. The questionnaire contains 36 items, representing health and non-health-related food characteristics, grouped into nine motivational dimensions, measuring the importance of “health”, “mood”, “convenience”, “sensory appeal”, “natural content”, “price”, “weight control”, “familiarity” and “ethical concern”. Participants were asked to indicate the importance of each of these 36 FCQ items for “the food I eat on a typical day” on a 4-point scale (1 = “not at all important” to 4 = “very important”). The nine motivational dimensions were computed as single scores for each dimension by calculating the mean of the unweighted items. 

#### 2.5.4. Satisfaction with Food-Related Life 

The satisfaction with food-related life (SWFL) questionnaire developed by Grunert et al. [26], evaluates cognitive judgements on an individual’s food-related life. It consists of 5 items grouped into a single dimension: (1) Food and meals are positive elements; (2) I am generally pleased with my food; (3) My life in relation to food and meals is close to ideal; (4) With regard to food, the conditions of my life are excellent; and (5) Food and meals give me satisfaction in daily life. Participants were asked to indicate their degree of agreement with each statement using a 5-point Likert scale (1—strongly disagree; 5—strongly agree). The SWFL score is the sum of the five items of the scale (range 5–25). Higher scores correspond with greater levels of SWFL. 

### 2.6. Statistical Analysis 

SPSS for MAC, version 26 (IBM Corp., 2019, Armonk, NY, USA), was used to analyse the data. Descriptive statistics were used for the demographic and clinical characteristics; the mean ± SD are presented for continuous data, the frequencies and percentages for categorical data, and the medians and interquartile range (IQR) for non-normally distributed data. BMI was calculated using the World Health Organization calculation (weight in kg divided by height in m2) [27]. Associations between variables were explored using chi-square tests for categorical data and a one-way analysis of variance for normally distributed continuous data. Mann–Whitney and Kruskal–Wallis tests were used for continuous data that were not normally distributed. SWFL score and DQS data were not normally distributed as tested by Shapiro–Wilk’s test and therefore non-parametric tests were utilised to investigate these variables. Pairwise comparisons were performed using Dunn’s (1964) procedure with a Bonferroni correction for multiple comparisons for all post-hoc tests. *p* < 0.05 was considered statistically significant. 

### 2.7. Thematic Analysis 

An optional open-ended question was included at the end of the questionnaire, giving participants the opportunity to add any more information on the topic. Free-text data from this question was analysed using Braun and Clarke’s updated six-phase process for conducting reflexive thematic analysis [28]. A six-phase process that systematically builds from data familiarization through to coding and theme development and refinement. Initially, a researcher (N.O.C.) independently read the text for data familiarization and generation of initial inductive codes. All research team members (N.O.C., L.K., and P.D.) reviewed and approved codes before applying to the transcripts. Microsoft Excel was used to manage the data as the sample size was not large enough to require specialised qualitative software. The coded data were analysed for initial themes by all team members. These themes were then refined and defined until the emergent four themes were agreed upon.

## 3. Results

### 3.1. Demographic and Clinical Characteristics

In total, 170 cancer survivors completed the online questionnaire. Most of the respondents were female (85.9%). The mean age was 51.5 ± 10.9 years (range 21–77 years). Over one third of the cohort were in full time employment and the majority (128, 75.6%) had some level of higher education. Breast cancer was the most common diagnosis (n = 110, 64.7%), followed by hematologic tumours (n = 19, 11.2%) and equally by gynecologic and testicular/prostate tumours (n = 11, 6.5%). Over half the cohort (n = 103, 60.1%) had completed treatment in the last five years. Completed treatments reported included chemotherapy (n = 130, 76.5%), surgery (n = 130, 76.5%), radiotherapy (n = 116, 68.2%) and hormone therapy (n = 60, 35.3%). Tamoxifen was the most common prescribed medication post-treatment (n = 56, 32.9%). Over half of the cohort were classified as overweight or obese (n = 95; 56.5%). Recent weight gain (last 6 months) was reported by 33.7% of the cohort (n = 57), with weight fluctuations being reported by an additional 22.9% (n = 39) (Table 1).

### 3.2. Dietary Score and Dietary Changes Post Cancer Treatment 

As displayed in Table 2, the mean dietary quality score for the cohort was 10.3 ± 1.7, with a range of 6–15, with 15 being the maximum achievable score. More than half the respondents (57.1%) reported a decrease in energy levels since completing treatment, while 65.3% and 64.1%, respectively, of the cohort reported their thirst and appetite remained the same. There was no significant difference between genders, age, or cancer type with regards to dietary quality score (Table 2).

The cohort reported dietary changes in terms of introducing (35.5%) or eliminating (40.8%) foods post-treatment. The most mentioned change was an increased intake of vegetables (n = 46, 27.1%). Red and processed meat consumption was reduced by the largest number of individuals (n = 45, 26.5%), followed by a reduction in sugar intake (n = 19, 11.1%).

#### Vitamin and Mineral Supplementation

Supplement use was reported by 69.8% (n = 118) of the cohort; 36.5% (n = 62) of the total cohort reported using a single supplement, 15.3% (n = 26) reported using two supplements and 16.8% (n = 30) reported using three or more supplements. The most popular choice of vitamin and mineral supplements among the cohort were vitamin D (45.5%), magnesium (18.8%), vitamin C (18.2%), calcium (14.7%) and a multivitamin (16.8%). There was no significant difference between genders, age, or cancer type with regards to supplement use (Table 2).

### 3.3. Food Choice

**Figure 1:**
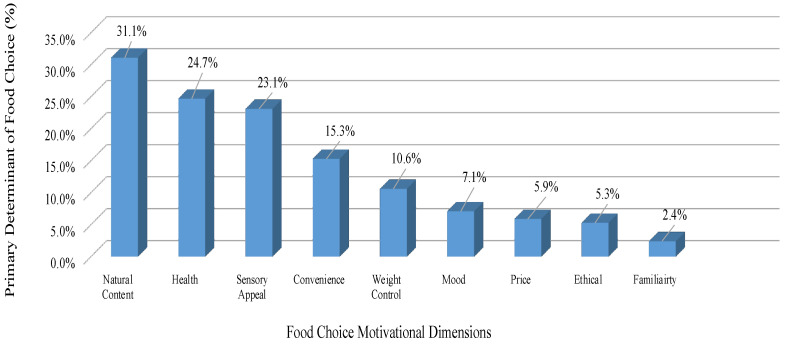
Primary food choice reported by the responders, in rank order.

Natural content was the primary determinant of food choice for the cohort (31.7%); the second most dominant was health at 24.7%, with 23.1% and 15.3% of the cohort indicating sensory appeal and convenience as their primary choice, respectively (Figure 1). 

### 3.4. Satisfaction with Food-Related Life 

The median (IQR) SWFL score for the cohort was 19 (3.3), ranging from 5 to 20, with a maximum possible score of 25. The sections “my life in relation to food and meals is close to ideal” and “with regard to food, the conditions of my life are excellent” received the lowest individual mean scores (Table 3).

#### Associations with SWFL and Age Category, BMI Categories and Supplement Use

The 36–50 years age category reported lower SWFL than those in the 65–80-year-old category (*p* = 0.007). The 36–50 years age category also had a lower SWFL than those in 18–36 years age category (*p* = 0.002). The 51–65 years age category reported lower SWFL than those in the 18–36-year-old category (*p* = 0.03). There was a significant difference between median SWFL scores between the healthy weight category and the obese category (*p* = 0.01), but not between any other group combination. There was a significant difference between the median SWFL scores for participants who took vitamin and mineral supplements (19.0 (4.0)) and those who did not (18.0 (4.0)) (Table 4).

### 3.5. Sources of Nutrition Information

#### 3.5.1. Nutritional Advice Received from a Dietitian

Most respondents (80%) reported that they had received no nutritional advice during their cancer treatment from a registered dietitian. Of those who did receive advice (20%), 7.1% were breast cancer survivors, 6.5% had haematological malignancies, 3.5% were head and neck cancer survivors, 1.8% had gynecologic cancers and 1.1% had upper gastrointestinal tract (UGI) cancers. Post-treatment, only 12% of our cohort received nutritional advice from a registered dietitian. Of this, half were breast cancer survivors (5.9%), 3.7% were head and neck cancer survivors and 2.4% had haematological malignancies.

#### 3.5.2. Nutritional Advice Received from Other Sources 

Overall, 22.4% (n = 38) of our cohort reported conducting self-directed research into nutrition or diet in cancer. This advice was acquired from different sources; of the total cohort, 10% (n = 15) reported carrying out their own online research mostly via social media, followed by 3.5% (n = 6) who received nutritional information at a cancer support centre and 4.8% (n = 8) who obtained information from a booklet or from an oncology consultant. The remaining 5.3% (n = 9) obtained information from an array of different sources classified as ‘other’, such as a personal trainer (n = 3), commercial diet programs (n = 2), friends (n = 2) or an acupuncturist (n = 2). 

### 3.6. Thematic Analysis 

In total, 30.5% (n = 52) of the total cohort responded to an optional open-ended question: “Would you like to say anything on the topic of nutrition practices among Irish cancer survivors?”. Four themes emerged.

*Theme 1: Desire for more specific nutritional advice and dietetic referral*
Respondents desire more advice through each stage of the disease process, diagnosis, treatment and post-treatment: “*I think nutritional advice and support should be provided during and after treatment. I was thrown into early menopause following treatment and only recently* *realised the effect hormones or lack of contribute to weight gain etc.”* (female, aged 41, cervical cancer). Several respondents noted the advice given is not specific to everyone: “*there is very little information available regarding dietary advice apart from ‘eat healthily’ and obviously one’s idea of what constitutes a healthy meal varies greatly from person to person*.” (female, aged 48, breast cancer). Cancer survivors in the present study were interested in learning and expanding their knowledge on nutrition; specifically, what foods to consume or avoid during and post cancer treatment: “*getting nutritional information after being diagnosed would have been good to give me ideas of what to avoid and what to add to my diet.*” (male, aged 45, liver cancer). Frequently, respondents voiced their desire for dietetic referral and stated it should be mandatory in oncology care: “*I think more help from dietitians should be standard of care for cancer patients. I had so many fluctuations in appetite and what I could/couldn’t stomach during treatment.”* (female, aged 67, colon cancer).
*Theme 2: Cancer and treatment nutrition impacts*
Many participants described the side effects of treatment they experienced. They expressed an interest in acquiring nutrition knowledge during treatment specifically on how to overcome or ease their treatment side effects (e.g., taste alterations, nausea, vomiting, loss of appetite and altered bowel habits): “*I would have liked more information during chemotherapy about my diet and coping with loss of taste.”* (female, aged 69, breast cancer): “*I am pleased to see the topic of appetite and eating being given attention as it is difficult to manage during and after treatment.”* (female, aged 49, breast cancer) and “*I have never had to consider my diet but since my treatment I have had to consider roughage and* *fibre as constipation is an ongoing issue—seven months on from treatment.”* (female, aged 23, lymphoma). One respondent explained that their relationship with their food has been impacted by their diagnosis and treatment: “*Since completing my treatment my relationship with food has totally changed. I now eat because I must, not because I want to. I vomit most every day which doesn’t help.”* (female, aged 52, breast cancer).
*Theme 3: Weight gain*
Several breast cancer survivors emphasized the struggle they have with weight gain since their diagnosis and treatment. One survivor mentioned never having previous struggles with weight gain: “*I struggle with my diet and weight since I was sick before that I’ve never gained weight.*” (female, aged 55, breast cancer). On returning to work, another breast cancer survivor highlighted the struggle she has with weight gain: “*Can be a bit of a struggle to try and keep my weight under control, since returning to work full time my weight has gradually increased*.” (female, aged 49, breast Cancer). Similarly, to our other themes, the emphasis on nutritional support throughout the cancer journey was highlighted in relation to weight gain: “*Nutritional advice and monitoring should be part of the cancer journey. I eat well exercise a lot but can’t shift the weight. I put on 2 stone 5 years ago and it won’t move*.” (female, aged 52, breast cancer)
*Theme 4: Interest in and awareness of nutrition*
In the final theme, respondents expressed their interest in nutrition and individual values: “*Nutrition is so important for cancer patients. More cancer patients die from malnutrition than cancer because they didn’t give their bodies the nutrients it needs because they lose their appetite. The body needs to be fueled with good food to work properly.”* (female, aged 49, breast cancer). It was stated as an important and interesting areas by respondents: “*an important area that I have no knowledge of*” (male, aged 51, lymphoma) and “*it’s an interesting topic, requires more attention.”* (female, aged 59, breast cancer). One participant stated they became more aware of nutrition post-treatment: *“such an interesting topic and something that I became so aware and interested about after treatment.”* (female, aged 23, leukaemia)

## 4. Discussion

Several key findings have arisen in this analysis of nutrition practices among cancer survivors who are post-treatment in Ireland. The mean dietary quality score was 10.3 ± 1.7 and median satisfaction with food-related life score was 19 (3.3). The primary determinant of food choice natural content (31.7%) followed by health (24.7%). Vitamin and mineral supplement use was reported by 69.8% of the cohort; the most consumed dietary supplement was vitamin D. To our knowledge, this study is the first of its kind to examine dietary quality, food choice and satisfaction with food-related life in cancer survivors across Ireland. 

In the literature, very few studies have compared the diet quality of cancer survivors to those without cancer [9,29,30]. In the few studies that have a lower post-diagnosis diet quality and poor adherence to dietary guidelines was present among cancer survivors [9,29,30]. In our study, dietary quality was assessed by a validated short-form food frequency questionnaire [24]; the mean dietary quality score of the cohort was 10.3 ± 1.7 and the range was 7–15. Dietary quality using this tool has not been assessed in cancer survivors, but it has been applied to the general population; for example, a large UK-based study had mean dietary quality of 11.4 ± 1.6, where a cut off score of 12 was applied for a healthy diet quality [24]. Both these studies had a modest diet quality score; however, our cohort were lower in comparison. Our cohort’s median SWFL score was 19 (3.3) (range 5–20), from a maximum possible score of 25. Likewise, this is lower than the general population study in central Chile, with an SWFL score of 22.9 ± 4.5 [31]. Overall, there are few studies for both DQS and SWFL which can be used as comparison. However, more robust measures of diet are more burdensome on the participants and these results now provides a baseline for Irish cancer survivors, which can be compared to moving forward. Additionally, we highlight the need for ongoing public health efforts to improve dietary intake, especially among cancer survivors, given they are high risk for secondary health problems [32]. 

Food choices are decided by a multitude of individual, social and environmental factors [33]. Natural content was the primary determinant of food choice for our cohort (31.7%). The second most dominant food choice for our cohort was health (24.7%). Perceptions of naturalness are associated with the degree to which foods are perceived to have been processed, with food that has undergone greater processing considered less natural [34]. These food choices were priority for the post-treatment survivors in our study, which may support the conventional belief that a diagnosis of cancer can be a “cue for action” that leads to positive dietary changes in survivorship [35]. Though, in several studies, participants reported that their cancer diagnosis had prompted them to make positive food choices, and the motivations for doing so are driven by beliefs about the importance of diet for improving general health [18,36]. To be noted, the following determinant in our study for food choice was sensory appeal (23.1%). Sensory appeal may be applicable in our cohort as taste changes can be inhibited due to the possible persistence of nutritional impact symptoms post-treatment; however, previous research has shown that taste alterations are transient, and usually recover within the three to six months after the end of chemotherapy [37,38]. 

A significant proportion of this cohort were breast cancer survivors (64.7%) who experienced post-treatment weight gain (27.1%) and weight fluctuations (15.3%). Weight gain is a profound issue among breast cancer patients and survivors [39]; it is associated with poor quality of life [40] and an increased risk of developing comorbid conditions [41]. Several factors may attribute to the weight gain in our cohort of breast cancer survivors: approximately half the breast cancer survivors received chemotherapy and hormonal therapy, and the majority were prescribed tamoxifen post-treatment. Often for breast cancer survivors, chemotherapy-associated weight gain is experienced during the first year after diagnosis [42]. The Women’s Healthy Eating and Living (WHEL) study found that breast cancer survivors treated with chemotherapy were 65% more likely to gain weight compared with those not receiving chemotherapy [43]. Additionally, research shows that women have decreased levels of physical activity after the diagnosis of breast cancer and 15% of breast cancer cases in postmenopausal women may be attributable to weight gain [44]. Our open-text results suggest that breast cancer survivors are highly concerned by weight gain and have a desire for guidance in an individual manner. Additionally, to be noted weight control was the fourth determinant of food choice for the cohort (10.9%). 

In the open-text responses, the desire for more specific nutritional advice and dietetic referral was repeated amongst cancer survivors. This desire was expressed for each stage of the disease process, diagnosis, treatment, and post-treatment, like many other Irish studies [16,17]. In this study, only 20% of survivors received nutritional advice during their cancer treatment from a registered dietitian. Post-treatment, only 12% of our cohort received nutritional advice from a registered dietitian. These results are lower than other Irish studies, where 26% and 39.4% of respondents received dietetic support, respectively [15,16]. This may be due to the high prevalence of breast cancer survivors in our cohort and often the limited dietetic services present are prioritized for more complex surgical cases within cancer centres. In Ireland, there are only 33 full-time equivalent dietitians with a remit in oncology, of which just 3 are clinical specialist dietitians, equating to a registered dietitian-to-patient ratio of 1:4500 [45]. 

Considering the low rate of nutritional support, it was foreseeable that over a fifth of the cohort conducted their own self-directed research on nutrition. The most common source of this research was carried out ‘online’ or on ‘social media’. Social media platforms, such as Facebook, Instagram and Twitter, and the online cancer support groups on these platforms, are an emerging source of social support in oncology. These platforms can be favorable in some regards by providing an avenue for patient engagement and empowerment, increasing informational support and relaying opportunities for clinical and research study participation [46]. Conversely, it can spread misinformation, overwhelm with information and expose survivors to financial exploitation [22]. This can lead to the information void being filled by unqualified and unreliable sources and alternative health providers promoting complementary and alternative medicine (CAM) [16]. Often patients may turn to alternative approaches such as supplement use and fad diets, often unsupported by scientific evidence [47]. A previous study found that 56% of Irish breast cancer patients used CAM, with 38% of the total cohort reporting using dietary interventions (taking antioxidants, health supplements, special diets, cleansing and high-dose vitamins [48]). 

The World Cancer Research Fund (WCRF) strongly encourages cancer survivors to obtain their nutritional needs through a healthy balanced diet alone, as opposed to taking supplements [49]. Conversely, in the literature, the use of vitamin and mineral dietary supplements among cancer survivors is widespread, with prevalence rates in breast, prostate and colorectal cancer survivors ranging from 50 to 85% [50,51]. Cancer survivors report a higher prevalence of using any (70.4% vs. 51.2%) and multivitamin/mineral (48.9% vs. 36.6%) supplement than individuals without cancer [51]. Similarly, prevalence rates were high in our study, with 69.8% of the cohort reporting vitamin and mineral supplement, with 17.6% of the cohort reporting using three or more supplements. In the literature, the most cited incentive for dietary supplement intake in cancer survivors is a high desire for personal control [52], to improve health (e.g., immune system) and prevent disease [53]. Given how widely available and accessible supplements are, a multitude of concerns exist, particularly since data are lacking regarding not only the effectiveness and quality control but also safety, especially in terms of possible drug interactions. It is essential for future studies to provide further evidence on the role of dietary supplement use post-treatment to develop evidence-based recommendations specially tailored for cancer survivors. 

Vitamin D was the most popular choice among the cohort, with 65.3% taking the supplement. The Institute of Medicine in the United States recommends 10 µg of vitamin D each day for the average person [54]. The Scientific Committee of the Food Safety Authority of Ireland recommends that the daily intake of vitamin D in older adults (aged 65 years and over) be 15 µg for those who are generally healthy and living independently, and 20 µg for those who are housebound or have limited sunlight exposure [55]. Recently, the Irish Joint Committee on Health launched a report on vitamin D deficiency, concluding that the Government must promote supplementation across the population by increasing public knowledge and reducing this supplement cost. An increase in the recommendation of vitamin D supplementation of 20–25 μg/day has been proposed for the entire adult population as a public health measure, where possible and where medically appropriate [56]. It should be noted that our cohort consisted of a large proportion of female participants, many breast cancer patients receiving hormone therapy and many postmenopausal women. These are all individuals at increased risk of osteoporosis; therefore, these factors might have impacted vitamin D supplement consumption.

A cancer diagnosis presents a potential teachable moment [29]; however, evidence-based information, guidance and support all need to be available to capitalise on this. In the present study, respondents reported post-dietary behaviours in terms of introducing (35.5%) or eliminating (40.8%) foods. The most mentioned dietary elimination was red processed meat and a reduction in sugar intake. In addition, an increase in vegetables, pulses, nuts and seeds were also reported. These post-treatment dietary changes are in line with the WCRF recommendations, which focus on aspects of diet, including following a dietary pattern rich in whole grains, vegetables, fruit, and beans, and limiting consumption of red meat and processed food, to reduce cancer incidence and mortality [49]. Similarly, in a qualitative study on the views on diet and cancer of cancer survivors’ in the United Kingdom [18], post-treatment changes were generally consistent with healthy eating recommendations, although dietary supplements and other non-evidence-based actions were mentioned. Likewise, in Sullivan’s national survey of oncology survivors, 37% of the cohort had tried alternative dietary strategies, including restrictive fad diets, herbal remedies, juicing, detoxes or they removed food groups such as meat, dairy and sugar, for fear of advancing their disease [16]. It is important that cancer survivors consult openly about removing food groups or the use of CAM with clinicians, to identify potentially harmful practices while having supportive dialogues about evidence-based measures [57]. 

This study had several limitations. This study’s cross-sectional design makes it impossible to determine cause from consequence. Furthermore, we did not ask about pre-diagnosis dietary habits except in the context of post-diagnosis dietary introductions or eliminations. Given the small and heterogeneous sample, it is difficult to draw firm conclusions about the absence or presence of any patterns based on participant characteristics, and we were not seeking to do so, rather to get an overview of current practices amongst a heterogeneous sample across Ireland. Furthermore, those who took part in our study may be those with a long-term interest in healthy lifestyles, or those who have become interested since diagnosis. We recruited through both online and by networking with cancer care centres across Ireland, meaning that some participants may be particularly motivated to find out information about their cancer and nutrition. As we recruited online, it is not possible to calculate a response rate to the study. As a final point, the data were collected during October to December 2020 where Ireland was in and out of ‘lockdown’ due the COVID-19 pandemic, and therefore, dietary quality and access to dietetic referral could have been impacted by the pandemic. 

## 5. Conclusions

Despite the stated limitations, this study provides useful insight into the nutrition practices of Irish cancer survivors. Survivors have a desire for individualized and specific advice relating to their nutrition problems—providing this could improve dietary quality and support weight management. There is a need for research into the nutrition-specific needs of cancer survivors and how best to deliver these needs to integrate nutrition into survivorship from a survivor perspective. It is imperative that the health service recognise the gaps in nutritional care in cancer survivorship and advocate for it as an integrated aspect of cancer care.

## Figures and Tables

**Table 1:** Summary of the demographic and clinical characteristics of the respondents to the survey (n = 170).

Respondents Characteristics	*n* (%) Mean ± SD
**Gender**	
Male	24 (14.1)
Female	146 (85.9)
**Age (years)**	51.5 ± 10.9
**Age group (years)**	
21–36	11 (6.5)
36–50	64 (37.6)
51–65	78 (45.9)
66–77	17 (10.0)
**Location**	
Republic of Ireland	155 (91.2)
Northern Ireland	15 (8.9)
**Education**	
Less than secondary school	6 (3.5)
Completed secondary school	29 (17.1)
Apprenticeship	7 (4.1)
PLC, diploma or certificate	53 (31.4)
Bachelor’s degree	46 (27.1)
Graduate degree (Master’s or PhD)	29 (17.1)
**Employment**	
Student	3 (1.8)
Part-time employment	27 (15.9)
Full-time employment	58 (34.1)
Sick leave	19 (11.2)
Homemaker	19 (11.2)
Self-employment	12 (7.1)
Unemployed	6 (3.5)
Retired	26 (15.3)
**Clinical Characteristics**	*n (%)*
**Primary tumour**	
Breast cancer	110 (64.7)
Haematological malignancies	19 (11.2)
Head and neck cancer	7 (4.1)
Testicular/prostate cancer	11 (6.5)
Gynecologic cancers	11 (6.5)
Other	12 (7.2)
**Years since treatment finished**	
1–2	53 (31.2)
3–5	49 (28.9)
6–10	45 (28.9)
>10	23 (13.8)
**Completed treatments**	
Chemotherapy	130 (76.5)
Radiotherapy	116 (68.2)
Hormone therapy	60 (35.3)
Surgery	130 (76.5)
**Medication prescribed post-treatment**	
Tamoxifen	56 (32.9)
Letrozole	10 (5.9)
Anastrozole	5 (2.9)
**BMI categories**	
Underweight (<18.5 kg/m^2^)	5 (2.9)
Healthy weight (18.5–24.9 kg/m^2^)	68 (40.5)
Overweight (25–29.9 kg/m^2^)	62 (36.9)
Obese (30–34.9 kg/m^2^)	33 (19.6)
**Weight Changes** **(within previous six months)**	
Weight gain	57 (33.7)
Weight loss	17 (10.0)
Weight fluctuations	39 (22.9)

**Table 2:** Nutritional characteristics, food changes and supplementation post-treatment.

Nutritional Characteristics	n (%)
**Dietary Quality Score (Mean ± SD)**	10.3 ± 1.7
**Appetite post-treatment**	
Increased	40 (23.5)
Decreased	21 (12.4)
Remained the same	109 (64.1)
**Thirst post-treatment**	
Increased	53 (31.2)
Decreased	6 (3.5)
Remained the same	111 (65.3)
**Energy levels post-treatment**	
Increased	36 (21.2)
Decreased	97 (57.1)
Remained the same	37 (21.8)
**Dietary changes**	**n (%)**
**Introduced**	
Yes	60 (35.5)
No	109 (64.5)
**Foods introduced**	
Fruit and vegetables	46 (27.1)
Pulses	14 (8.3)
Nuts/seeds	9 (5.4)
Fish	3 (1.8)
Protein	2 (1.2)
**Removed**	
Yes	69 (40.8)
No	109 (64.5)
**Foods removed**	
Red/processed meat	45 (26.5)
Reduced sugar	19 (11.1)
Diary	6 (3.6)
Fried food	2 (1.2)
Processed food (high sodium)	7 (4.1)
**Vitamin and mineral supplementation**	**n (%)**
**Supplement intake**	
Yes	118 (69.8)
No	51 (30.2)
**Number of supplements**	
1	62 (36.5)
2	26 (15.3)
3	15 (8.8)
4	10 (5.9)
5	5 (2.9)
**Type of supplement**	
Vitamin D	77 (45.5)
Magnesium	32 (18.8)
Vitamin C	31 (18.2)
Calcium	25 (14.7)
Multi-Vit	23 (13.5)
Omega 3	17 (10.0)
Probiotic	9 (5.3)
Zinc	7 (4.1)

**Table 3:** Median score of five items of the Satisfaction with Food Life (SWFL) scale.

Variables	Median (IQR)
Food and meals are positive elements	4.0 (1.0)
I am generally pleased with my food	4.0 (0.0)
My life in relation to food and meals is close to ideal	3.0 (1.0)
With regard to food, the conditions of my life are excellent	3.0 (1.0)
Food and meals give me satisfaction in daily life	4.0 (1.0)
Overall SWFL	19 (3.3)

**Table 4:** Associations between the median (IQR) SWFL score and age category, BMI categories and supplement use.

	SWFL Median (IQR)	*p*-Value
**Age Categories**		
18–36 years	21 (2.0)	0.001 *
36–50 years	18 (4.0)	
51–65 years	19 (3.0)	
65–80 years	20 (8.0)	
**BMI categories**		
Underweight (<18.5 kg/m^2^)	16 (14.0)	0.001 **
Healthy weight (18.5–24.9 kg/m^2^)	19.5 (3.0)	
Overweight (25–29.9 kg/m^2^)	19.0 (18.0)	
Obese (30–34.9 kg/m^2^)	18.0 (3.0)	
**Supplement use**		
Yes	19.0 (4.0)	0.001
No	18.0 (4.0)	

* Post-hoc comparisons using the Bonferroni correction indicates level of significance between those in the 36–50 years age category and 65–80 years age category (*p* = 0.007), between individuals in the 36–50 years age category and the 18–36 years age category (*p* = 0.002) and the 18–36 years age category and the 51–65 years age category (*p* = 0.03). ** Post-hoc comparisons using the Bonferroni correction indicates level of significance between those median SWFL scores between the healthy weight category and the obese category (*p* = 0.01).

## Data Availability

The data presented in this study are available on reasonable request from the corresponding author.

## Supplementary Materials

The following supporting information can be downloaded at: https://www.mdpi.com/article/10.3390/nu14040767/s1, File S1: Questionnaire.
