# Supplementary material for: Nutrition Practices among Adult Cancer Survivors Living on the Island of Ireland: A Cross-Sectional Study
Source: Nutrients. 2022 Feb 11;14(4):767. doi: 10.3390/nu14040767 (PMC8880791; doi:10.3390/nu14040767)
Supplement: Supplementary file 1 [file nutrients-14-00767-s001.zip › nutrients-1557241-supplementary.pdf]

NUTRICO - Nutrition Related Life of Irish Cancer Survivors

The aim of this study is to evaluate the current dietary intake, food choices and satisfaction with food related life in Irish cancer survivors. This is phase one of an Irish Research Council funded masters research project.

* Required

Section 1 - Participant Information Leaflet & Consent Form

There is a growing number of cancer survivors in Ireland (200,000 in 2020) and the demand to recognise the individual and distinct needs of cancer survivors has been highlighted by both the National Registry through recent scoping work and the government through the development of a National Cancer Strategy 2017-2026. It has been recognized that not enough attention has been given to the long-term consequences of a cancer diagnosis or as to how to enable individuals to return to active lives following the completion of initial cancer treatment.

There are no data available on the dietary intake, supplement use or residual nutrition impact symptoms in Irish cancer survivors. In this online study, we would like to gather information on the nutritional status of cancer survivors, so we can better determine and inform the need for nutrition support in this area.

Who is organizing and funding this study?

This study is part of a master's research project being undertaken by Miss Niamh O'Callaghan under the supervision of Ms. Laura Keaver of the Department of Health and Nutritional Science at Institute of Technology Sligo and Ms. Pauline Douglas of the Nutrition Innovation Centre for Food and Health (NICHE) at Ulster University. This research study is being funded by the Irish

Research Council.

Why am I being asked to take part?

You are eligible to participate in the study if you have completed active cancer treatment at least six months ago.

Is the study confidential?

All information you provide will be treated with strict privacy and confidentiality at all time and will not be identified in the published findings. Although there is no direct benefit from participating in this research, findings which emerge from this study may potentially enhance nutrition and dietetic support in the oncology setting.

You don't have to take part in this study, your participation is voluntary, you can change your mind about taking part in the study and leave by exiting the browser. None of your data will be stored or used. Once you click submit you will be unable to withdraw as your information will not be identifiable.

1. Please tick below, if you consent to participate in this study.
   If you would not like to participate, please exit the browser. *

   ○ I consent to participate in this study.

Section 2 - Demographic

2. To which gender do you most identify? *

- Male
- Female
- Prefer not to say

3. What age are you? *

4. What height are you? *

5. What is your current weight? Please state if you use kg or stone/pounds. *

6. Where is you home located? *

- Republic of Ireland
- Northern Ireland
- Other

7. What is your current living situation? *

- I live alone
- I live with my partner/family/relatives
- Other

8. What is the highest level of school you have completed, or the highest degree received? *

- Less than secondary school
- Completed Secondary School
- Apprenticeship
- PLC, Diploma or cert
- Bachelor's degree
- Graduate school degree (Master's or PhD)

9. What is your current employment status? *

- ◯ Full-time employment
- ◯ Full-time employment (sick leave)
- ◯ Part-time employment
- ◯ Part-time employment (sick leave)
- ◯ Self-employment
- ◯ Self-employment (sick leave)
- ◯ Unemployed
- ◯ Home-maker
- ◯ Student
- ◯ Retired

10. Have you previously received any of the following (Tick all that apply) *

- ☐ Chemotherapy
- ☐ Chemotherapy and radiotherapy
- ☐ Radiotherapy
- ☐ Hormonal Therapy
- ☐ Surgery
- ☐ Other

11. What cancer(s) type were you diagnosed with? *

12. What year were you diagnosed with cancer(s)? *

13. When did you finish cancer treatment? Please state the year. *

14. Have you been prescribed any medication since finishing your cancer treatment?

15. Since completion of cancer treatment, have you experienced any of the following?

- [ ] Fatigue
- [ ] No appetite
- [ ] Nausea
- [ ] Vomiting
- [ ] Dry mouth
- [ ] Food/drinks taste funny or have no/little taste
- [ ] Problems swallowing
- [ ] Mouth sores
- [ ] Smells bother you
- [ ] Pain
- [ ] Constipation
- [ ] Diarrhoea
- [ ] ______

Other

16. If you ticked yes to experiencing any of the above, please tick which of these you have experienced in the last 6 months?

- [ ] Fatigue
- [ ] No appetite
- [ ] Nausea
- [ ] Vomiting
- [ ] Dry mouth
- [ ] Food/drinks taste funny or have no/little taste
- [ ] Problems swallowing
- [ ] Mouth sores
- [ ] Smells bother you
- [ ] Pain
- [ ] Constipation
- [ ] Diarrhoea
- [ ] Other

17. Have you experienced weight changes in the last 6 months? *

- [ ] I have experienced weight loss
- [ ] I have experienced weight gain
- [ ] I have experienced weight fluctuations (both weight loss and weight gain)
- [ ] I have remained the same weight

18. During your cancer treatment, did you see a registered dietitian to receive nutritional advice? *

- ◯ Yes
- ◯ No

19. Since completing treatment, have you seen a registered dietitian to receive nutritional advice? *

- ◯ Yes
- ◯ No

20. Since completion of your cancer treatment, have you received any nutritional advice from any source other than a dietitian? *

- ◯ Yes
- ◯ No

21. If you answer yes to the previous question, can you elaborate on where you received the nutritional advice from?
(i.e. from a doctor, nurse, online, books, information booklet, social media, friend, family member)

22. Since completion of your cancer treatment, has your appetite *

- Increased
- Remained the same
- Decreased

23. Since completion of your cancer treatment', has your thirst *

- Increased
- Remained the same
- Decreased

24. Since completion of your cancer treatment, have your energy levels *

- ◯ Increased
- ◯ Remained the same
- ◯ Decreased

Section 3 - Food Frequency Questionnaire

25. The following questions ask about some foods & drinks you might have during a ‘typical’ week, over the past month or so. Do not be concerned if some things you eat or drink are not mentioned.

Please tick how often you eat at least ONE portion of the following foods & drinks: (a portion includes: a handful of grapes, an orange, a serving of carrots, a side salad, a slice of bread, a glass of pop).

Please only put one tick, but answer EVERY line *

	Rarely of Never	Less than 1 a week	Once a week	2-3 times a week	4-6 times a week	1-2 times a day	3-4 times a day
Fruit (tinned / fresh)	○	○	○	○	○	○	○
Fruit juice (not cordial or squash)	○	○	○	○	○	○	○
Salad (not garnish added to sandwiches)	○	○	○	○	○	○	○
Vegetables (tinned / frozen / fresh but not potatoes)	○	○	○	○	○	○	○
Chips / fried potatoes	○	○	○	○	○	○	○
Beans or pulses like baked beans, chickpeas, dahl	○	○	○	○	○	○	○
Fibre-rich breakfast cereal, like Weetabix, Fruit ‘n Fibre, Porridge, Muesli	○	○	○	○	○	○	○

Wholemeal bread or chapattis	○	○	○	○	○	○	○
Cheese / yoghurt	○	○	○	○	○	○	○
Crisps / savoury snacks	○	○	○	○	○	○	○
Sweet biscuits, cakes, chocolate, sweets	○	○	○	○	○	○	○
Ice cream / cream	○	○	○	○	○	○	○
Non-alcoholic fizzy drinks or sugar sweetened berverage (not sugar free or diet)	○	○	○	○	○	○	○

26. The following questions ask about some meats/fish you might have during a 'typical' week. Do not be concerned if some things you eat or drink are not mentioned.

Please tick how often you eat at least ONE portion of the following foods (a portion includes: 50-75 grams of cooked beef, lamb or chicken (half the size of the palm of your hand), 100 grams of fish.
If you do not eat meat or fish, please tick rarely/never.

- please only put one tick, but answer EVERY line *

	Rarely or never	Less than 1 a week	Once a week	2-3 times a week	4-6 times a week	7+ times a week
Beef, Lamb, Pork, Ham - steaks, roasts, joints, mince or chops	○	○	○	○	○	○
Chicken or Turkey – steaks, roasts, joints, mince or portions (not in batter or breadcrumbs)	○	○	○	○	○	○

Sausages, bacon, corned beef, meat pies/pasties, burgers	○	○	○	○	○	○
Chicken/turkey nuggets/ turkey burgers, chicken pies, or in batter or breadcrumbs	○	○	○	○	○	○
White fish in batter or breadcrumbs – like ‘fish ‘n chips’	○	○	○	○	○	○
White fish not in batter or breadcrumbs	○	○	○	○	○	○
Oily fish – like herrings, sardines, salmon, trout, mackerel, fresh tuna (not tinned tuna)	○	○	○	○	○	○

27. Have you introduced any new foods to your diet since completing your cancer treatment? *

○ Yes

○ No

28. If yes, can you elaborate:

29. Have you removed any food from your diet since completing your cancer treatment? *

- ◯ Yes
- ◯ No

30. If yes, can you elaborate:

31. Do you take any vitamins, minerals or any other food supplements? *

- ◯ Yes
- ◯ No

32. If yes, can you elaborate:

Section 4 - Factors that influence your choice of food

Several different factors influence our choice of food. For every person, there will be a different set of factors that is important. In the next set of questions, we are interested in finding out what factors influence your choice of food.
Listed below are a series of factors that may be relevant to your choice of foods. Read each item carefully and decide how important the item is to you. Put a tick in the box that best reflects your feelings. Remember, there are no right or wrong answers - we are interested in what is important to you.

33. It is important to me that the food I eat on a typical day:

	Not important at all	A little important	Moderately important	Very important
is easy to prepare	○	○	○	○
contains no additives	○	○	○	○
is low in calories	○	○	○	○
tastes good	○	○	○	○
contains natural ingredients	○	○	○	○
is not expensive	○	○	○	○
is low in fat	○	○	○	○
is familiar to me	○	○	○	○
is high in fibre and roughage	○	○	○	○
is nutritious	○	○	○	○
is easily available in shops	○	○	○	○
is good value for money	○	○	○	○

cheers me up	◯	◯	◯	◯
smells nice	◯	◯	◯	◯
can be cooked very simply	◯	◯	◯	◯
helps me cope with stress	◯	◯	◯	◯
helps me control my weight	◯	◯	◯	◯
has a pleasant texture	◯	◯	◯	◯
is packaged in an environmentally friendly way	◯	◯	◯	◯
comes from countries I approve of politically	◯	◯	◯	◯

34. It is important to me that the food I eat on a typical day:

	Not important at all	A little important	Moderately important	Vert important
is like the food I ate when I was a child	◯	◯	◯	◯
contains lots of vitamins and minerals	◯	◯	◯	◯
contains no artificial ingredients	◯	◯	◯	◯
keeps me awake and alert	◯	◯	◯	◯
looks nice	◯	◯	◯	◯
helps me relax	◯	◯	◯	◯

is high in protein	○	○	○	○
takes no times to prepare	○	○	○	○
keeps me healthy	○	○	○	○
is good for my teeth/skin/hair/ nails etc.	○	○	○	○
makes me feel good	○	○	○	○
has the country of origin clearly marked	○	○	○	○
is what I usually eat	○	○	○	○
helps me to cope with life	○	○	○	○
can be bought in shops close to where I live or work	○	○	○	○
is cheap	○	○	○	○

Section 5 - Satisfaction with Food-related Life

35. Listed below are factors contributing to satisfaction with food-related life. Read each statement carefully and decide how important the item is to you. Put a tick in the box that best reflects your feelings. Remember, there are no right or wrong answers - we are interested in what is important to you. *

	Strongly Disagree	Disagree	Neutral	Agree	Strongly agree
Food and meals are positive elements.	○	○	○	○	○
I am generally pleased with my food.	○	○	○	○	○
My life in relation to food and meals is close to ideal.	○	○	○	○	○
With regard to food, the conditions of my life are excellent	○	○	○	○	○
Food and meals give me satisfaction in daily life.	○	○	○	○	○

36. Would you be interested in discussing this topic further and would you be happy for us to contact you to take part in focus groups around this topic? *

○ Yes

○ No

37. Please leave your contact details below, this information will not be used for any other purpose and will be deleted once used. *

38. Is there anything else you would like to say about this topic?

39. Before you submit your response, has COVID-19 or the current restrictions impacted you in the areas of food or nutrition? *

◯ Yes

◯ No

40. Can you please elaborate? *

12/10/2021

This content is neither created nor endorsed by Microsoft. The data you submit will be sent to the form owner.

Microsoft Forms
